# *Ab initio* engineering of materials with stacked hexagonal tin frameworks

**DOI:** 10.1038/srep28369

**Published:** 2016-07-08

**Authors:** Junping Shao, Clément Beaufils, Aleksey N. Kolmogorov

**Affiliations:** 1 Department of Physics, Applied Physics and Astronomy, Binghamton University, State University of New York, Binghamton, New York 13902-6000, USA

## Abstract

The group-IV tin has been hypothesized to possess intriguing electronic properties in an atom-thick hexagonal form. An attractive pathway of producing sizable 2D crystallites of tin is based on deintercalation of bulk compounds with suitable tin frameworks. Here, we have identified a new synthesizable metal distannide, NaSn_2_, with a 3D stacking of flat hexagonal layers and examined a known compound, BaSn_2_, with buckled hexagonal layers. Our *ab initio* results illustrate that despite being an exception to the 8-electron rule, NaSn_2_ should form under pressures easily achievable in multi-anvil cells and remain (meta)stable under ambient conditions. Based on calculated *Z*_2_ invariants, the predicted NaSn_2_ may display topologically non-trivial behavior and the known BaSn_2_ could be a strong topological insulator.

The morphology of a crystal structure reflects the underlying bonding mechanism and gives indications about material’s potential applications. For instance, the high atomic density and symmetry of the *sp*^3^-connected framework make the diamond polymorph of carbon the hardest known bulk substance^1^. The pronounced anisotropy of graphite arising from the weak van der Waals interlayer binding allows for material’s intercalation with different metals and creation of excellent alkali-ion battery electrodes^2^. The rigid, naturally hole-doped *sp*^2^ boron backbone in magnesium diboride is responsible for the material’s record critical temperature in the class of ambient-pressure phonon-mediated superconductors^3^. The reduced dimensionality of free-standing *sp*^2^ frameworks of carbon determines an array of unique properties, from exceptionally high carrier mobilities^4^ to the the quantum Hall effect at room temperature^5^. Making ‘carbon’ copies of such Nature’s best structural motifs can be an effective strategy in the search for materials with even more appealing properties.

Group-IV elements are a natural choice for creating novel (meta)stable covalent networks due to the automatic fulfilment of the 8-electron rule in structures with well-defined bonding and anti-bonding states^6^. The ability of carbon to form extended *sp*^2^ nanostructures is credited to the additional *π*-bonding which strengthens the hexagonal sheets. For all other tetravalent elements the diamond configuration is favored over the layered morphology. Moreover, the favorability of the *sp*^2^ or *sp*^3^ hybrid bonding diminishes with the element’s number due to the reduction of bond integrals^7^. Consequently, tin readily transforms from the semiconducting diamond *α*-phase to the metallic higher-coordinated *β*-phase near room temperature while lead adopts the typical metallic fcc structure.

Reflective of this stability trend within group IV, graphene was the first material to be produced as a free-standing atom-thick sheet, with silicene^8^,^9^ and germanene^10^ synthesized later as single layers deposited on metallic surfaces. Zhu *et al*. have just reported on the first successful synthesis of stanene via molecular beam epitaxy on a Bi_2_Te_3_ substrate^11^. The present effort to produce free-standing stanene has been inspired, in part, by the prediction of the material’s behavior as a large-gap quantum spin Hall insulator induced by a strong spin-orbit coupling (SOC)^12^. Due to an unwanted influence of the substrate on the material’s electronic properties^11^ and evident thermodynamic instability of 2D tin relative to the bulk phases (see Fig. 1), alternative synthesis routes need to be explored to produce desired free-standing tin-based polymorphs. Halogenation has been proposed as a possible pathway to creating stable variants of stanene while preserving its exotic electronic features^13^,^14^, but 2D tin sheets are still a required precursor. Recalling that exfoliation of graphite was the original route to graphene and that sheets of germanane have been obtained through topochemical deintercalation of CaGe_2_ with HCl^15^, we have carried out an *ab initio* search for new synthesizable bulk compounds comprised of hexagonal tin layers. In addition to being possible precursors, such compounds may display topologically non-trivial electronic properties or superconductivity observed previously in related materials comprising graphene-like layers^3^,^16^,^17^ or belonging to the Zintl family^18^.

*Ab initio* structure prediction has been playing an increasingly important role in materials development and already led to discovery of new materials^19^,^20^,^21^. Successful computational strategies for identifying new synthesizable materials range from automated screening with high-throughput^22^,^23^ or machine learning^24^ techniques to targeted search with unconstrained optimization^19^,^25^,^26^ or rational design^27^ methods. The exploration of a large configurational space of structures and compositions in this study has been driven primarily by the latter, physics-based *ab initio* material engineering approach. Having established beneficial features for stability of the desired structural motif, we homed in on the Na-Sn binary system in which a new NaSn_2_ phase is predicted to form under compressions as low as a few GPa as discussed on page 5, remain (meta)stable under ambient conditions, and, to the best of our knowledge, feature the first all-tin stacking of flat hexagonal layers.

A great variety of stable hexagonal layered structures - differing in the number or valence of the constituent elements as well as in the layer morphology or stacking order (see Fig. 1) - have been observed or predicted in previous studies. The AlB_2_ prototype (hP3 in the Pearson notation, Fig. 2a) is the basic recurring 3-atom structure from which all the other layered variants can be derived. For example, metal diborides have been observed to have not only flat but also buckled B layers^19^. The known hP3-LiBC^28^ has hexagonal nets based on B and C, the predicted^29^ and recently observed^30^ hP8-Li_2_B_2_ has an extra close-packed Li layer inserted between the B sheets, while the recently discovered hP8-Na_2_MgSn has a composite Mg-Sn hexagonal layer *and* an extra Na layer^31^. BaSn_2_ appears to be the only synthesized compound with zigzag Sn layers^32^.

## Computational Methods

All our calculations were performed at the density functional theory (DFT) level with the VASP code^33^,^34^. We used projector-augmented potentials^35^ with the 4*d* electrons of Sn and 2*p* electrons of Na treated as semi-core. We chose a high energy cut-off of 500 eV and fine *k*-meshes to ensure numerical convergence of relative energies to within 1–2 meV/atom. The final results were obtained with the exchange-correlation functional parameterized by Perdue, Burke, and Ernzerhof^36^ within the generalized gradient approximation (GGA) but we checked the sensitivity of our results to systematic errors by performing additional calculations within the local density approximation (LDA)^37^ and the non-local van der Waals density functional optB86b^38^. We found that SOC had an insignificant effect on relative stability. The vibrational energy contribution to the Gibbs energy calculated within the frozen phonon method^39^,^40^,^41^, on the other hand, was shown to be an noticeable stabilization factor for the proposed phase. The parity analysis was carried out with QUANTUM ESPRESSO package^42^. Further technical details can be found in the Supplementary Information.

## Results and Discussion

The primary goal of this study was to determine whether stable Sn-based compounds could form in the simplest hP3 configuration. As the first step, we calculated the formation energy of hP3-*MA*_2_ compounds where *A* was C, Si, Ge, Sn, or Pb and *M* was one of 41 common metals (see Supplementary Information). According to the results of the systematic scan in Fig. S1, all the C-based compounds have positive formation energy, i.e., the smallest-size C networks do not benefit from the insertion of a metal layer. The larger tetravalent elements are seen to have islands of stability for metals with up to 5 electrons in the outer shells. For *M*Sn_2_, they are Li, Na, K, Mg, Ca, Sr, Ba, Sc, Y, La, and Zr, as seen in Fig. 3a. The negative formation energy with respect to the ground state elemental structures is a necessary but not sufficient condition for compounds’ thermodynamic stability. The required construction of the convex hull under select (*P*, *T*) conditions involves a considerable effort for systems rich in complex phases. In order to narrow down our search, our next step was to analyze the importance of the electron count and the size of the added metal on the compound’s stability.

Figure 4 shows the evolution of the band structure and DOS as a binary compound is assembled from single flat Sn^2D^ sheets by stacking the layers up into a Sn^3D^ framework and then intercalating it with a metal layer. The Dirac-type crossings of the *p*_*z*_ states at the *K* and *H* points (in blue) serve as a good marker for the relative position and population of *s*-*p*-derived bands. In an ideal case of degenerate *s* and *p* starting atomic orbitals (Δ*E*_*sp*_ = 0 eV), the valence *sp*^2^-hybridized *σ* bands would have the 1:2 ratio of *s* and *p*_*xy*_ characters and be separated from the corresponding conduction set by a large gap. The remaining electrons would fill all the bonding *π* states ensuring the placement of the Dirac point exactly at the Fermi level. Even though Δ*E*_*sp*_ in all tetravalent elements is considerable (7.5 ± 1 eV 7), the model gives an adequate description of graphene.

The orbital hybridization mechanism in Sn^2D^ is quite different. The bands in the (−9.6, −7.4) eV and the (−3.3, −1.1) eV ranges show little mixing and can be considered as bonding *s*^+^ and 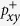, respectively. The antibonding *s*^−^ states are now high enough in energy to hybridize partially with the second set of the *p*_*xy*_ states, as was discussed for stanene by Xu *et al*.^14^. The resulting bonding *s*^−^*p*_*xy*_ hybrid states extend from −7.4 to just above −1.8 eV while the antibonding ones begin at −1.1 eV. The partial occupation of the latter drains electrons from the bonding *p*_*z*_ states and leaves the Dirac crossings about 0.4 eV above the Fermi level.

**Figure 2:**
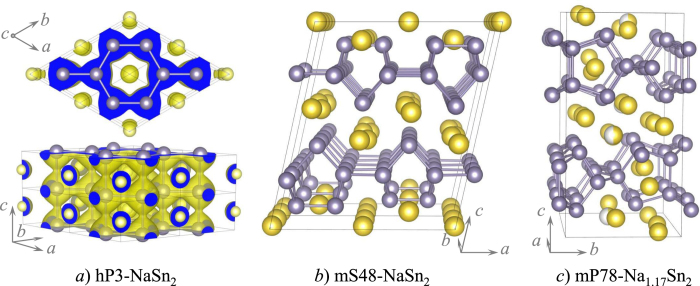
Predicted (**a**) and known (**b**,**c**) binary compounds of Na (large yellow) and Sn (small grey). The charge density isosurface in (**a**) illustrates comparable intra- and interlayer overlaps of Sn orbitals. The 4 yellow-white spheres in (**c**) are Na sites with 0.5 occupancy.

The relative band positions change considerably once the sheets are AA-stacked. The appearance of two additional neighbors only 3.11 Å away along the *c*-axis disperses the *p*_*z*_ band along the Γ − *A* direction by nearly 10 eV. Interpretation of the band structure becomes more difficult because the *s*^−^ states get involved in the formation of the *s*^−^*p*_*z*_ *σ* bonds as well [the comparable strengths of the covalent bonding within and between the layers can be seen from the charge density distribution in Fig. 2a.] The outcome of the change in bands’ position and degree of mixing is the appearance of a new key feature: a deep DOS minimum at 1.3 eV above *E*_Fermi_. The amount of charge in the checkered area needed to fill up the bonding states is close to 0.5 electron (or 1.0 e for 2 Sn atoms). Based on this analysis, monovalent metals should be particularly good candidates to stabilize the Sn^3D^ structure.

Figure 4c shows that addition of Na indeed moves the Fermi level near the bottom of the DOS minimum. The charge transfer from the Na 5*s* states 5 eV above *E*_Fermi_ does not follow the rigid band approximation. The positive potential of Na^+^ downshifts the energy of the states derived from *s* and *p*_*xy*_ the most because they extend farthest towards the metal site. Hence, the charge is transferred primarily onto the bonding *s*^−^*p*_*xy*_ hybrid states.

**Figure 3:**
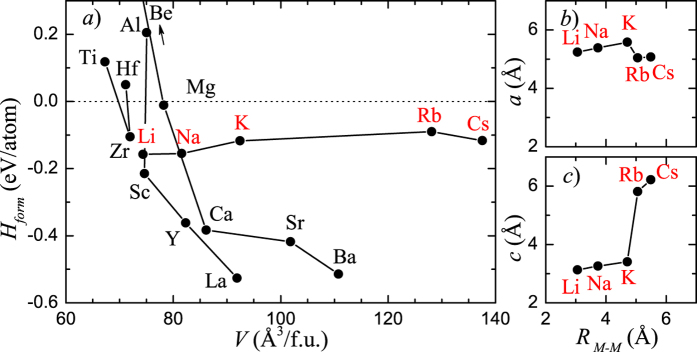
(**a**) Calculated formation energies for AlB_2_-type *M*Sn_2_ compounds grouped by *M*’s electron count. (**b**,**c**) Variation of the *a* and *c* lattice constants in the AlB_2_-type monovalent metal stannides as a function of the alkali metal nearest neighbor distance in fcc.

**Figure 4:**
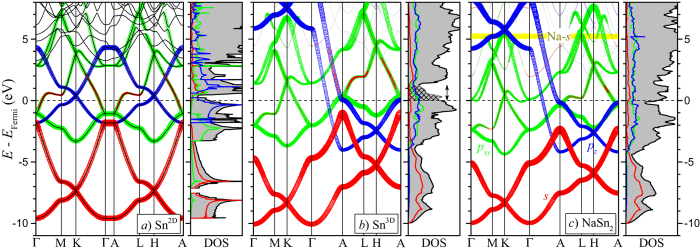
Calculated band structures and densities of states (DOS) without SOC for hypothetical (**a**,**b**) and predicted (**c**) materials with hexagonal flat frameworks: (**a**) a single Sn layer; (**b**) an AA-type stacking of Sn layers; (**c**) an AlB_2_-type NaSn_2_ compound. The size of the red, green, and blue circles is proportional to the character of the Sn *s*, *p*_*xy*_, and *p*_*z*_ states, respectively. The checkered DOS area in (**b**) contains approximately 0.5 electrons. The full DOS bar is 2.0 states/(eV u.c. spin) for the unit cells (u.c.) containing 2, 2, and 3 atoms in (**a**–**c**), respectively.

In order to see which of the alkali metals has the best chance of stabilizing the structure we examined the importance of the metal size factor. Figure 3c shows that the steady increase of the *M*Sn_2_ cell volume in the Li-Cs series masks an important irregularity occurring between K and Rb. For the larger Rb, the in-plane Sn-Sn distance shrinks but only because of the considerable expansion of the *c*-axis (see Fig. 2a). The interlayer *σ* bonds are effectively severed when the larger alkali atoms push the layers apart to above 5.5 Å. The loss of a major part of the 0.4-eV/atom covalent interlayer binding can be partly compensated by the layer buckling. However, a calculation of the *M*Sn_2_ relative stability with respect to the known alkali metal stannides indicated that only Na has a chance to be thermodynamically stable under near-ambient conditions.

Our next set of calculations was dedicated to the stability of hP3-NaSn_2_ relative to the known ambient-pressure compounds in this binary system. The existence of mS48-NaSn2,^43^ a direct reference point at the 1:2 composition (Fig. 2b), simplified the task. However, we also calculated the formation energy of relevant known structures in the full Na_*x*_Sn_1−*x*_ range to examine to which extent the compounds at nearby stoichiometries could affect the synthesizability of hP3-NaSn_2_ under considered (*P*, *T*) conditions.

The Na-Sn binary system has been shown to have complex compounds some of which have been discovered in recent years^44^,^45^,^46^. On the Na-rich side, the oS52-Na_9_Sn_2_ phase with isolated Sn polyanions and cI76-Na_30_Sn_8_ phase with isolated tin atoms are correctly reproduced as low-temperature ground states. The phases with *x* ≤ 0.5 include tI64-NaSn, tP12-NaSn_5_, and oS288-Na_40_Sn_104_ phases with complex 3D Sn frameworks. The last two compounds are 7 meV and 23 meV above the tie-line indicating their possible metastability.

**Figure 5:**
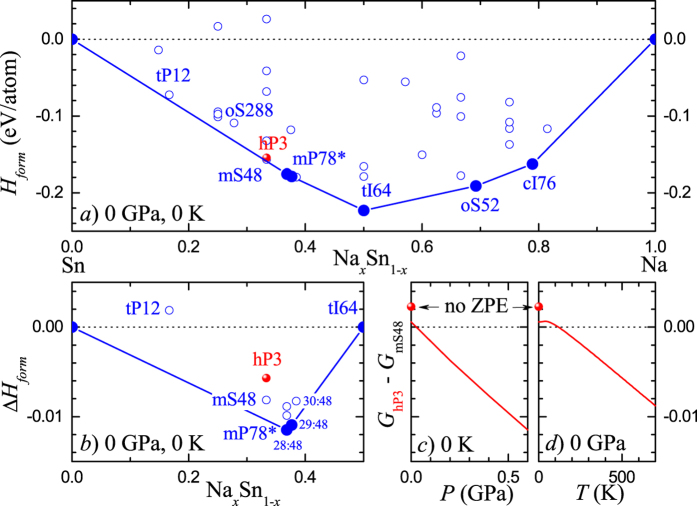
Calculated stability of Na-Sn compounds. (**a**) Formation energies calculated at 0 GPa and 0 K. (**b**) Relative formation enthalpies of Sn-rich compounds with respect to Sn and NaSn. The solid blue circles in (**a,b**) correspond to previously reported compounds found to be thermodynamically stable in our calculations. (**c**,**d**) Gibbs energy difference as a function of (*P*, *T*) illustrating stabilization of the predicted hP3-NaSn_2_ over the known mS48-NaSn_2_; Fig. S2 shows hP3-NaSn_2_ to be thermodynamically stable with respect to *all* considered compounds at 10 GPa. Zero point energy (ZPE) is taken account only in (**c**,**d**). All energy units in the figure are eV/atom.

The most relevant for the present study compounds are located at or near the 1:2 composition and covered by the Zintl rule^46^. The compound with the mP78 configuration (Fig. 2c) comprises of polyanion layers stacked up along the *c*-axis 9.0 Å apart^47^. Each layer in mP78 consists of 3- or 4-fold coordinated Sn atoms, arranged in 5-member rings covalently bonded along the *b* direction to form empty channels across the *a* direction. The 4-fold coordinated Sn atoms form the backbone of the layer while the lone pairs of the 3-coordinated Sn are stabilized by their proximity to the Na cations. Partial occupation of some of the Na sites in mP78 leads to an unusual Na_1.17_Sn_2_ stoichiometry and was indeed found in our study (see Supplementary Information) to be beneficial for compound’s stability: Fig. 5b shows ground states at two compositions near mP78. Removal of certain Na atoms generates mS48-NaSn_2_ (Fig. 2b). This 1:2 phase is slightly metastable in our calculations at 0 K and its formation could in reality be determined by a kinetic route starting from the closely related Na_1.17_Sn_2_.

The proposed hP3-NaSn_2_ is 2.3 meV/atom above mS48 but could be stabilized by several factors. Zero point energy (ZPE) alone makes it virtually degenerate with mS48 in Gibbs energy at 0 K while the vibrational entropy corrections at elevated temperatures or the *PV* enthalpic term at relatively low pressures make it the true ground state (Figs 5a,c,d and S2). The accuracy of the DFT approximations does not allow us to reliably define the (*P*, *T*) boundaries of the hP3 stability region. In the LDA, which tends to overstabilize denser structures, hP3 (*V*_G*GA*_ = 27.1 Å/atom) is already more stable than mS48 (*V*_G*GA*_ = 30.3 Å/atom) at 0 K (no ZPE) by 18 meV/atom. Dispersive interactions can be a factor defining the ground state not only in covalent graphitic structures^48^ but also in ionic-covalent materials^49^. In the case of hP3 the Sn-Sn interlayer spacing is considerably smaller compared to that in mS48 and the optB86b non-local functional indeed favors the former by 5.6 meV/atom. Similar trends in relative stability are expected with respect to the closely related, more complex mP78^*^ phases: pressure, in particular, destabilizes the whole set uniformly (see Fig. S2). Our phonon calculations show absence of imaginary modes in hP3 (see Supplementary Information).

**Figure 6:**
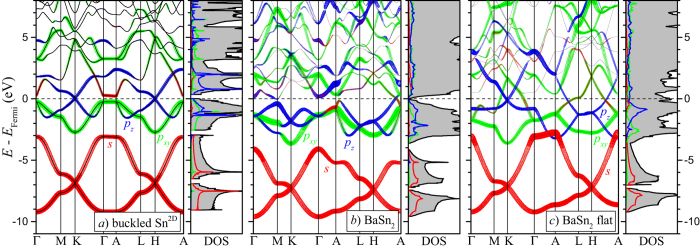
Calculated band structures and densities of states (DOS) without SOC for (**a**) a hypothetical single zigzag Sn layer; (**b**) a known BaSn_2_ compound with zigzag Sn layers; and (**c**) a hypothetical BaSn_2_ compound with flat Sn layers. The color coding of state characters is the same as in Fig. 4.

While alkali metals appear to be the preferred intercalants for the stacked flat layers of Sn, more electron-rich metals may stabilize buckled layers. Stable structures with zigzagged Sn and Ge layers have indeed been observed in BaSn_2_^32^ and CaGe_2_^15^, both of the EuGe_2_ type (space group number 164), which makes the closest chemical analog, SrSn_2_, a promising candidate. According to our calculations, SrSn_2_ misses stability by only 11 meV/atom (Fig. S3) with respect to the known hR48-SrSn_3_ and oS32-Sr_3_Sn_5_^50^. Bandstructure plots for the stable BaSn_2_ and the metastable SrSn_2_ phases (Figs 6 and S5), reveal that layer buckling is an alternative route to place the Fermi level near the bottom of a DOS minimum. Since the large Ba and Sr atoms push the Sn layers far apart (so that they assume the most stable single-layer zigzag configuration) it might be possible to disassemble the compounds into 2D networks completely by means of electrochemical deintercalation, thermal degassing, or other methods^15^,^51^,^52^. Our preliminary calculations indicate that compared to the observed reaction between HCl and CaGe_2_ producing 2D GeH and CaCl_2_, the similar reaction between HCl and BaSn_2_ would be less favorable by about 1.7 eV per *MA*_2_ formula unit but still exothermic.

**Figure 7:**
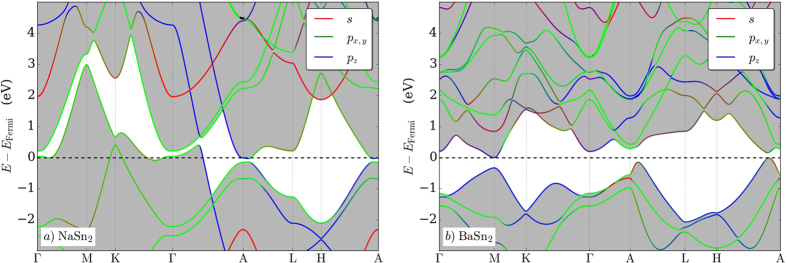
SOC-induced separation of bands into two groups in the predicted NaSn_2_ and the known BaSn_2_ materials by 0.1 eV and 0.18 eV, respectively.

In order to investigate the potential of these bulk Sn-based materials to exhibit topologically non-trivial behavior we examined the effect of spin-orbit coupling (SOC) on their electronic features, using well-established methods for materials with inversion symmetry^53^,^54^. In NaSn_2_, the splitting due to this relativistic effect does separate electronic bands into two groups, albeit by only a 0.1 eV gap (see Fig. 6). Evaluating the parity eigenvalues for the valence bands in the bulk NaSn_2_ structure at time-reversal invariant momenta (see Supplementary Information), we find that the *Z*_2_ invariants are 0;(001) which indicates that the compound is ‘weakly’ topological^55^. However, preliminary slab calculations reveal that creation of either Na- or Sn-terminated 001 surfaces does not result in bridging the gap with just the desired surface states: breaking the interlayer Sn-Sn bond brings additional *p*_*z*_ bands to the Fermi level. In BaSn_2_ , the SOC splits the band crossing between H and A points by 0.18 eV. The parity calculation demonstrates that this metal distannide has *Z*_2_ invariants *ν*_0_;(*ν*_1_*ν*_2_*ν*_3_) = 1;(001). Even though the band structure plot in Fig. 7 shows a zero indirect band gap the compound is not necessarily a semi-metal due to limitations of the standard DFT approximations. A non-zero bulk band gap could be observed in more accurate treatment of the excited states or obtained via doping in future studies. If a true band gap can be achieved the material will be the first strong topological insulator based on a layered Sn material.

## Conclusions

Our *ab initio* calculations show that hP3-NaSn_2_ should be at least metastable under ambient conditions and synthesizable in multi-anvil cells under relatively low pressures. The origin of the hP3-NaSn_2_ stability can be traced to the appearance of a deep minimum in the DOS of the Sn^3D^ layered framework. Na has the suitable size and the right valence to fit into the framework and fill some of the available bonding *s*^−^*p*_*xy*_ hybrid states. The 0.4-eV covalent interlayer bonding is essential for stabilization of the compound but will make it difficult to exfoliate the material. The relative position of electronic bands, defined by the *c*/*a* ratio and the presence of Na^+^ cations, is difficult to predict beforehand. The final band structure has the *p*_*z*_ Dirac points far away from *E*_Fermi_, −2.6 eV below and 5.8 eV above it. However, the successful identification of a synthesizable compound with hexagonal Sn layers gives grounds for optimism that related stable materials with different compositions, stacking orders, etc., could be derived in future studies. Based on an *ab initio* examination of bulk electronic states, the known BaSn_2_ material has the potential to be a strong topological insulator. A previous systematic search for superconducting features in C, Si, Ge-based AlB_2_-type compounds revealed a number of candidates materials with critical temperatures of about 10 K^56^. The large gradient of the DOS near *E*_Fermi_ in the metallic hP3-NaSn_2_ prohibits us from making a reliable estimate of the material’s *T*_*c*_. The widely used methods for calculating the electron-phonon coupling assume a constant DOS near *E*_Fermi_ which would lead to dramatic errors in this case. However, the DOS value 1.2 exactly at *E*_Fermi_ is higher than 1.0 state/(eV u.c. spin) in *β*-Sn and it would not be surprising if synthesized hP3-NaSn_2_ displayed comparable superconducting properties.

## Additional Information

**How to cite this article**: Shao, J. *et al*. *Ab initio* engineering of materials with stacked hexagonal tin frameworks. *Sci. Rep*. **6**, 28369; doi: 10.1038/srep28369 (2016).

## Supplementary Material

Supplementary Information

## Figures and Tables

**Figure 1:**
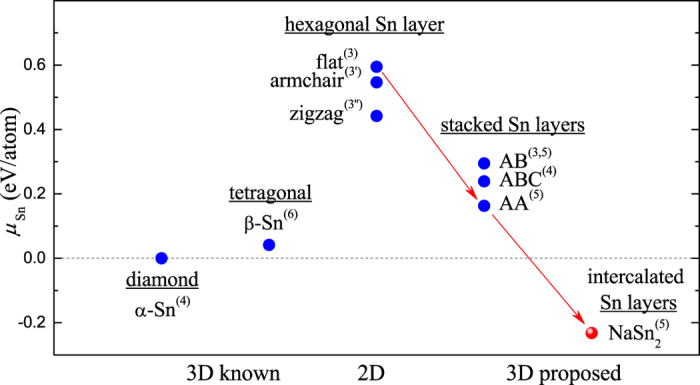
Calculated chemical potential of Sn in known 3D, hypothetical 2D/3D, and the predicted 3D Sn-based structure. The arrows illustrate the construction of the predicted hP3-NaSn_2_ out of flat hexagonal Sn layers. The superscripts show the coordination numbers for Sn atoms.
