# Supplementary material for: Ab initio engineering of materials with stacked hexagonal tin frameworks
Source: Sci Rep. 2016 Jul 8;6:28369. doi: 10.1038/srep28369 (PMC4937409; doi:10.1038/srep28369)
Supplement: Supplementary Information [file srep28369-s1.pdf]

Supplementary Information for: *Ab initio* engineering of compounds with stacked hexagonal tin frameworks

Junping Shao, Clément Beaufils, and Aleksey N. Kolmogorov
[1] *Department of Physics, Applied Physics and Astronomy, Binghamton University, State University of New York, PO Box 6000, Binghamton, New York 13902-6000, USA*
(Dated: June 17, 2016)

I Computational details
Total energy calculations 1
Structure library 1
Phonon calculations 2

II Tables
Calculated structural parameters of selected Na-Sn phases 2
Parities and parity eigenvalues for the calculation of Z_2 invariants in $NaSn_2$ 2
Parities and parity eigenvalues for the calculation of Z_2 invariants in $BaSn_2$ 3

III Figures
S1 Formation energy of MX_2 compounds at 0 GPa 3
S2 Formation enthalpy of Na-Sn phases at 0 and 10 GPa 4
S3 Formation Gibbs energy of Sr-Sn phases 4
S4 Band structures for α-Sn, β-Sn and hP3-$NaSn_2$ 5
S5 Band structures for flat 2D-Sn, $EuGe_2$-type $SrSn_2$ and AlB_2-type $SrSn_2$. 5
S6 Band structure for hP3-$NaSn_2$ with and without spin-orbit coupling. 6
S7 Band structure for hP3-$BaSn_2$ with and without spin-orbit coupling. 6

IV References 7

Total energy calculations

We used VASP [1] for all density functional theory (DFT) calculations. The final results of the relative stabilities were obtained with the Perdew-Burke-Ernzerhof (PBE) exchange-correlation (xc) functional [2] within the generalized gradient approximation (GGA) [3]. We used PAW potentials [4] with relevant semi-core electrons included, e.g., $4d$ electrons for Sn and $2p$ electrons for Na. The 500 eV energy cutoff and dense k-meshes, $N_{k1} \times N_{k2} \times N_{k3} \times N_{atoms} \geq 4,000$ [5], ensure numerical convergence of the formation energy differences to typically within 1-2 meV/atom. For example, a Γ-centered $12 \times 12 \times 18$ mesh was required to converge the energy of hP3-$NaSn_2$. We found that the spin-orbit coupling had negligible effect (under 1 meV/atom) on the relative stability of the $NaSn_2$ polymorphs.

To see the possible systematic errors due to the approximated form of the xc functionals we calculated relative energies for key phases using the Perdew-Zunger parameterization within the local density approximation (LDA) [6] and optB86b parameterization of with the van der Waals corrections [7]. As discussed in the manuscript, the difference in the relative energies was considerable. hP3-$NaSn_2$ was further stabilized with respect to the less compact mS48-$NaSn_2$ in the two DFT treatments. The results are consistent with the general tendency of LDA and van der Waals functionals to favor more compact structures [8].

Structure library

We considered about 40 relevant prototypes known at ambient pressure for metal stannides, in particular, for alkali and alkaline-earth metals. The 41 metals considered in this work are listed in our previous study[9]. We also ran evolutionary searches with MAISE[10] for 1:2 and 1:1 Na:Sn compositions up to 4 formula units using settings described in our previous studies [11, 12]. The most stable phases at 10 GPa were found to be hP3 and mP6 (see Table 1).

For mP78-$Na_{1.17}Sn_2$ (see Fig. 2) we simulated the partial occupancy of one Na $4g$ Wyckoff site by removing one or two atoms in the conventional unit cell. The resulting phases with the 0.75 and 0.50 fractional occupancies and the corresponding 29:48 and 28:48 stoichiometries indeed gained in stability at $T = 0$ K (see Fig. 5b). Among the three non-equivalent u6 at the 28:48 composition, the one with furthest separation of vacancies was found to be the most stable. Configurational entropy serves as an additional stabilization factor at finite temperatures. Even if one

assumed equal energies for the different decorations[13], the Gibbs energy would be lowered by less then 30 meV/atom at $T = 500$ K in this case.

Phonon calculations

We calculated the corrections to $G(T)$ due to the vibrational entropy in Fig. 5c,d with the finite displacement method as implemented in PHON [14]. We used finer $N_{k1} \times N_{k2} \times N_{k3} \times N_{atoms} \geq 6,000$ k-meshes and optimized cell parameters to match stresses within 0.1 kB and reduce forces below 0.01 eV/Å. The size of the displacement was set to 1/40 Å. The phonon density of states were found for sufficiently large supercells: $4 \times 4 \times 4$ for hP3-$NaSn_2$ and $4 \times 4 \times 3$ for mS48-$NaSn_2$ with $4 \times 4 \times 4$ and $4 \times 4 \times 3$ k-meshes, respectively.

Composition	Pressure (GPa)	Pearson symbol	Space group	a (Å)	b (Å)	c (Å)	Wyckoff positions			
								Sn		Na
NaSn	10	mP6	10 (P12/m1)	5.4295	3.1289	7.6976	1a	(0, 0, 0)	2n	(0.2963, $\frac{1}{2}$, 0.3296)
							2m	(0.2051, 0, 0.6605)	1e	($\frac{1}{2}$, $\frac{1}{2}$, 0)
$NaSn_2$	0	hP3	191 (P6/mmm)	5.3782		3.2464	2c	($\frac{1}{3}$, $\frac{2}{3}$, 0)	1b	(0, 0, $\frac{1}{2}$)
$NaSn_2$	0	mS48	12 (C2/m)	13.663	6.9835	15.637	8j	(0.4537, 0.2146, 0.2529)	4g	(0, $\frac{1}{4}$, 0)
							8j	(0.6711, 0.2831, 0.2528)	4i	(0.2716, 0, 0.0892)
							8j	(0.3477, 0.2818, 0.3962)	4i	(0.9144, 0, 0.5842)
							4i	(0.8843, 0, 0.1149)	4i	(0.3785, 0, 0.5671)
							4i	(0.6733, 0, 0.1145)		

TABLE I: Pearson symbols, space groups, lattice parameters and Wyckoff positions of fully relaxed Na-Sn structures.

i	TRIM	$m = 1$	2	3	4	5	6	7	8	9	10	11	12	13	14	15	16	17	18	19	δ_i
1	(0, 0, 0)	+1	-1	-1	-1	+1	+1	-1	-1	+1	+1	-1	-1	-1	+1	+1	-1	+1	+1	-1	(+1)
2	(0, 0, 0.5)	- 1	+1	+1	+1	+1	-1	-1	+1	+1	-1	+1	+1	-1	-1	+1	-1	-1	+1	+1	(+1)
3	(0, 0.5, 0)	+1	-1	-1	-1	-1	+1	-1	+1	-1	+1	-1	+1	-1	+1	-1	+1	-1	+1	-1	(-1)
4	(0, 0.5, 0.5)	-1	+1	+1	+1	-1	+1	-1	+1	-1	+1	-1	+1	-1	+1	-1	+1	+1	-1	-1	(-1)
5	(0.5, 0, 0)	+1	-1	-1	-1	-1	+1	-1	+1	-1	+1	-1	+1	-1	+1	-1	+1	-1	+1	-1	(-1)
6	(0.5, 0, 0.5)	-1	+1	+1	+1	-1	+1	-1	+1	-1	+1	-1	+1	-1	+1	-1	+1	+1	-1	-1	(-1)
7	(0.5, 0.5, 0)	+1	-1	-1	-1	-1	+1	-1	+1	-1	+1	-1	+1	-1	+1	-1	+1	-1	+1	-1	(-1)
8	(0.5, 0.5, 0.5)	-1	+1	+1	+1	-1	+1	-1	+1	-1	+1	-1	+1	-1	+1	-1	+1	+1	-1	-1	(-1)

TABLE II: Parity eigenvalues $\xi_{2m}(\Gamma_i)$ and parities δ_i at eight time-reversal invariant momenta (TRIM) for $NaSn_2$. We consider even bands up to $m = 19$ below the observed gap, indicated in FIG. 6 in the main article. The calculated Z_2 topological invariants are $\nu_0; (\nu_1\nu_2\nu_3) = 0; (001)$.

i	TRIM	$m=1$	2	3	4	5	6	7	8	9	10	11	12	13	14	15	16	17	18	19	δ_i
1	(0, 0, 0)	+1	+1	+1	-1	-1	+1	-1	+1	-1	+1	-1	-1	-1	-1	+1	-1	+1	+1	+1	(-1)
2	(0, 0, 0.5)	-1	+1	+1	-1	-1	+1	-1	+1	-1	+1	-1	+1	+1	+1	+1	-1	+1	-1	+1	(+1)
3	(0, 0.5, 0)	+1	-1	+1	-1	+1	-1	+1	-1	+1	-1	+1	-1	-1	-1	-1	+1	+1	-1	-1	(-1)
4	(0, 0.5, 0.5)	-1	-1	+1	-1	+1	-1	+1	-1	+1	+1	-1	+1	+1	+1	-1	+1	-1	+1	-1	(-1)
5	(0.5, 0, 0)	+1	-1	+1	-1	+1	-1	+1	-1	+1	-1	+1	-1	-1	-1	-1	+1	+1	-1	-1	(-1)
6	(0.5, 0, 0.5)	-1	-1	+1	-1	+1	-1	+1	-1	+1	+1	-1	+1	+1	+1	-1	+1	-1	+1	-1	(-1)
7	(0.5, 0.5, 0)	+1	-1	+1	-1	+1	-1	+1	-1	+1	-1	+1	-1	-1	-1	-1	+1	+1	-1	-1	(-1)
8	(0.5, 0.5, 0.5)	-1	-1	+1	-1	+1	-1	+1	-1	+1	+1	-1	+1	+1	+1	-1	+1	-1	+1	-1	(-1)

TABLE III: Parity eigenvalues $\xi_{2m}(\Gamma_i)$ and parities δ_i at eight time-reversal invariant momenta (TRIM) for $BaSn_2$. We consider even bands up to $m = 19$ below the observed gap, indicated in FIG. 6 in the main article. The calculated Z_2 topological invariants are $\nu_0; (\nu_1\nu_2\nu_3) = 1; (001)$, indicating that $BaSn_2$ could be a strong topological insulator.

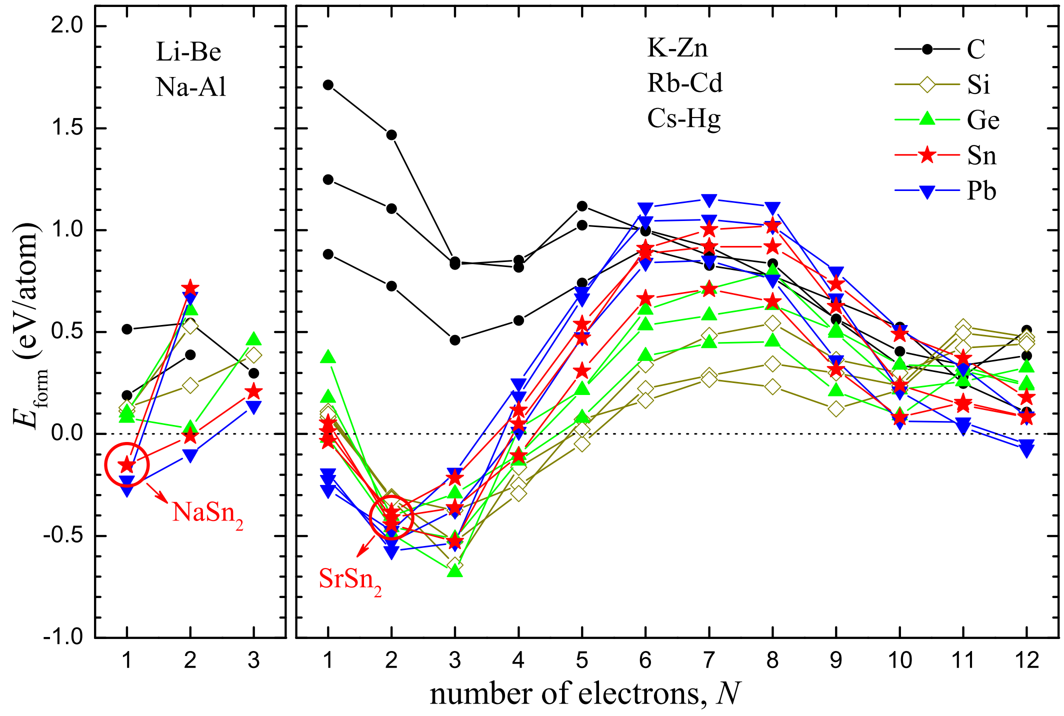

FIG. S1: Formation enthalpies for MX_2 where X = C, Si, Ge, Sn, Pb and M are 41 common metals. The points are plotted versus the metals' electron count and grouped according to their position in the periodic table.

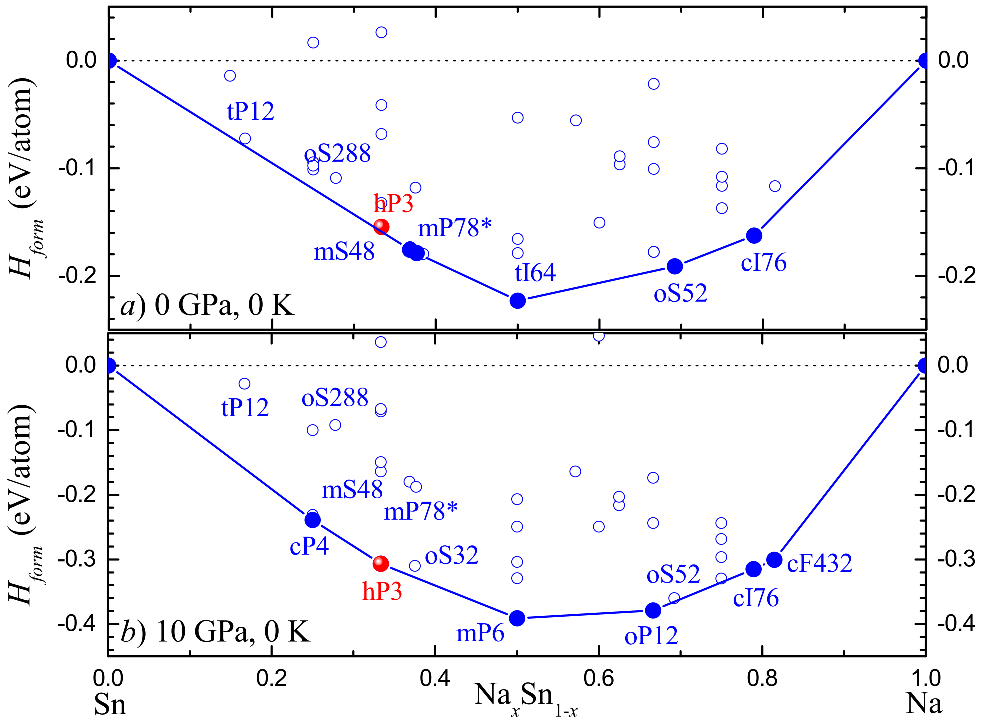

FIG. S2: (color online) Calculated formation enthalpies for Na-Sn phases at 0 and 10 GPa. The reported phases at ambient pressures are labeled; however, some of them were found to be metastable. The non-stoichiometric mP78∗ phase was simulated as described in the text above. The predicted hP3 phase with the AlB_2 prototype is expected to be the ground state at pressures of a few GPa.

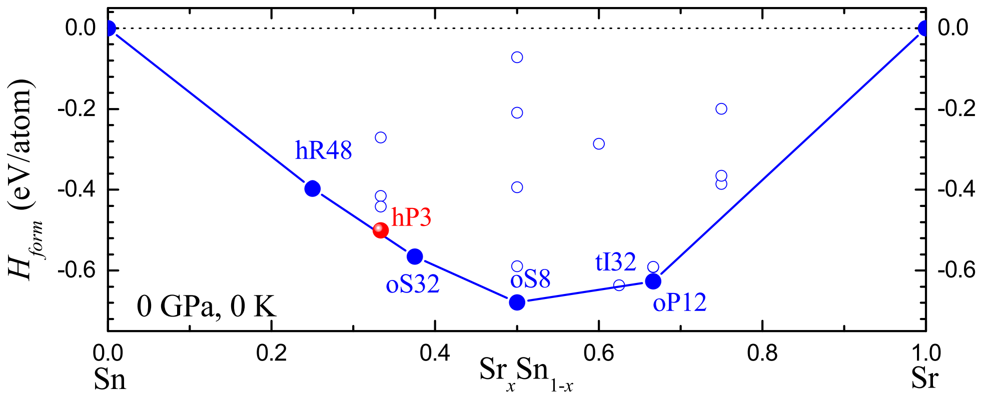

FIG. S3: (color online) Calculated stability of Sr-Sn compounds. Formation energies calculated at 0 GPa pressure. hP3-$SrSn_2$ is found to be 11 meV/atom above the tie-line between neighboring stable compounds and is destabilized at high temperature and pressure.

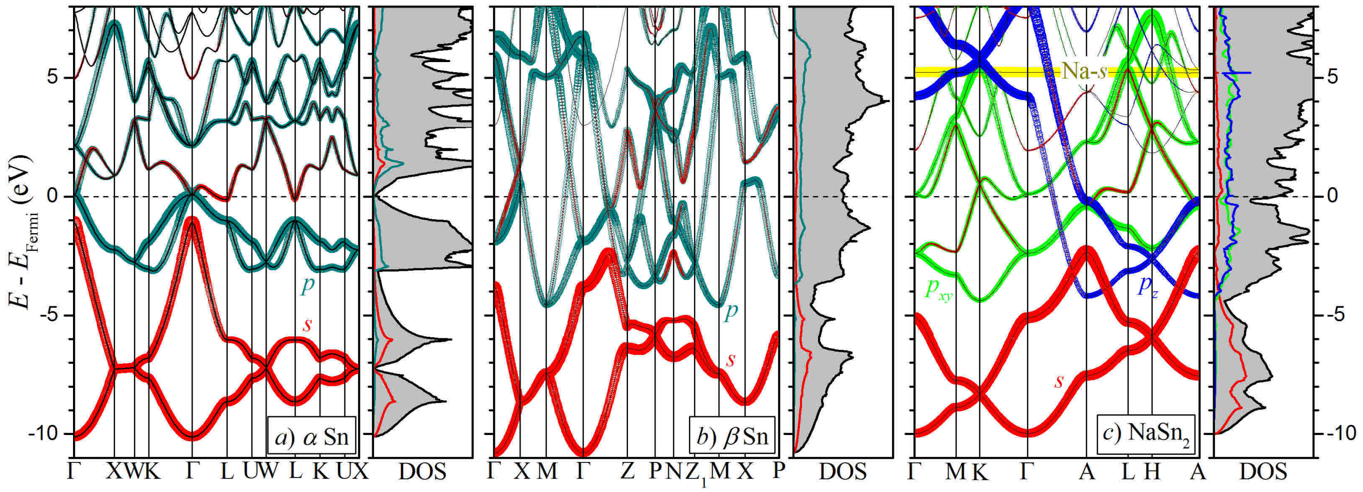

FIG. S4: (color online) Calculated band structures and densities of states (DOS) for (a,b) elemental tin and (c) $NaSn_2$: (a)α-Sn; (b)β-Sn; (c) an AlB_2-type $NaSn_2$ compound. The size of the red, green, blue, and dark cyan circles is proportional to the character of the Sn s, p_{xy}, p_z, and p states, respectively. The checkered DOS area in (b) contains approximately 0.5 electrons. The full DOS bar is 2 states/(eV u.c. spin).

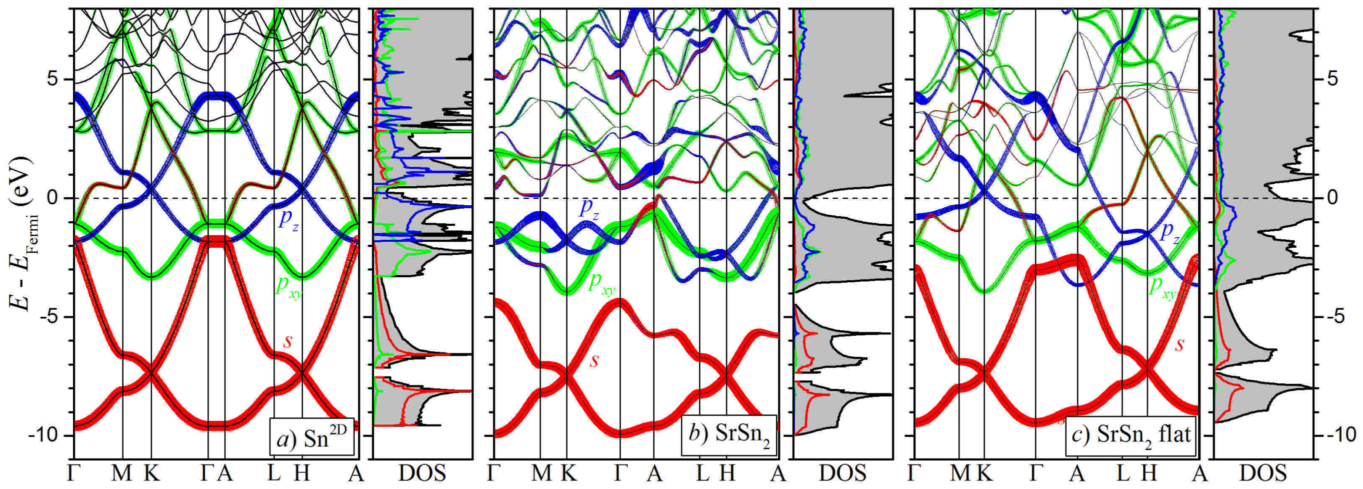

FIG. S5: (color online) Calculated band structures and densities of states (DOS) for (a) flat 2D tin and (b,c) two forms of AlB_2-type compound $SrSn_2$: (a)flat 2D-Sn; (b)buckled hP3-$SrSn_2$, prototype $EuGe_2$ (space group no. 164); (c) flat hP3-$SrSn_2$, prototype AlB_2 (space group no. 191). The size of the red, green, and blue circles is proportional to the character of the Sn s, p_{xy}, and p_z states, respectively. The full DOS bar is 2 states/(eV u.c. spin).

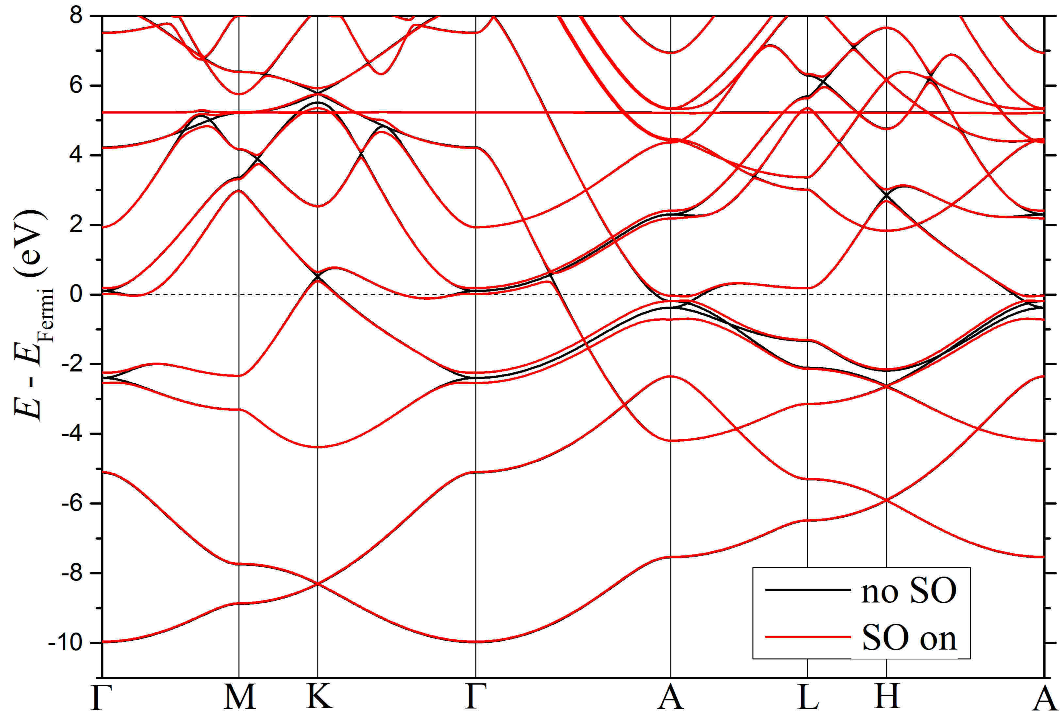

FIG. S6: (color online) Calculated band structures for $NaSn_2$ with (red lines) and without (black lines) spin-orbit coupling.

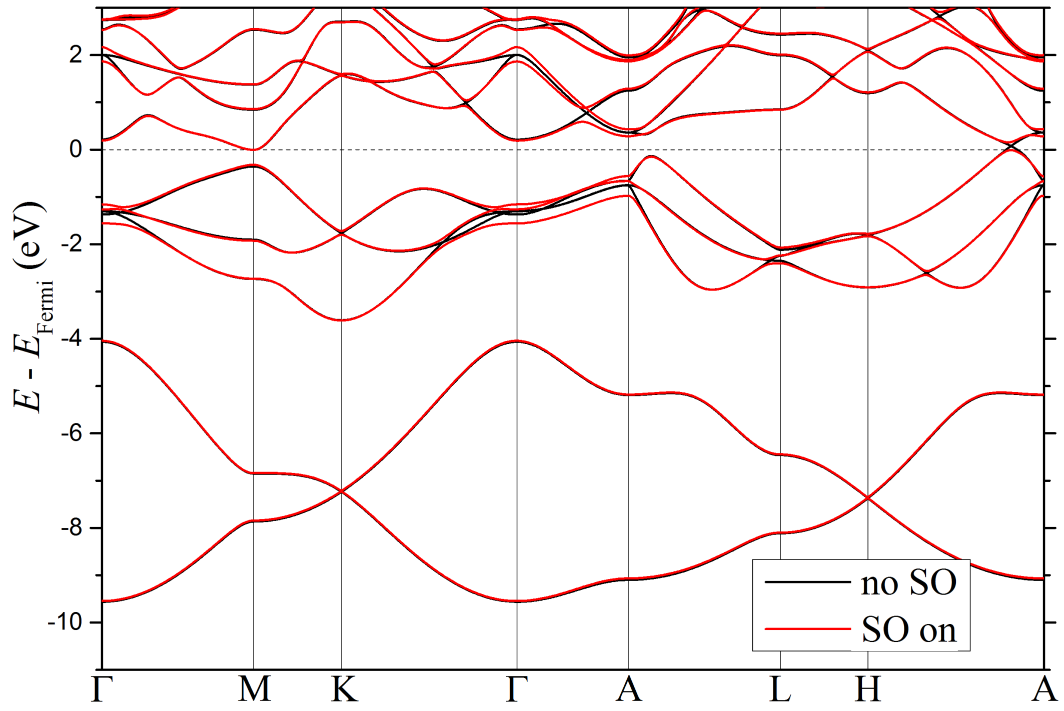

FIG. S7: (color online) Calculated band structures for $BaSn_2$ with (red lines) and without (black lines) spin-orbit coupling.

[1] G. Kresse and J. Hafner, Phys. Rev. B **47**, 558 (1993); G. Kresse and J. Furthmüller, Phys. Rev. B **54**, 11169 (1996).
[2] J.P. Perdew, K. Burke, and M. Ernzerhof, Phys. Rev. Lett. **77**, 3865 (1996).
[3] J.P. Perdew, K. Burke, and M. Ernzerhof, Phys. Rev. Lett. **78**, 1396 (1997).
[4] P.E. Blöchl, Phys. Rev. B **50**, 17953 (1994).
[5] J. D. Pack and H. J. Monkhorst, Phys. Rev. B **13**, 5188 (1976); **16**, 1748 (1977).
[6] J.P. Perdew, and A. Zunger, Phys. Rev. B **23**, 5048 (1981).
[7] J. Klime, D.R. Bowler, and A. Michaelides, J. Phys.: Condens. Matter **22**, 022201 (2010).
[8] A. Bil, B. Kolb, R. Atkinson, D. G. Pettifor, T. Thonhauser, and A. N. Kolmogorov. van der waals interactions in the ground state of $Mg(BH_4)_2$ from density functional theory. *Physical Review B*, 83(22):224103–, 06 2011.
[9] A. G. Van Der Geest and A. N. Kolmogorov. Stability of 41 metal–boron systems at 0 GPa and 30 GPa from first principles. *CALPHAD*, 46:184–204, 9 2014.
[10] A. N. Kolmogorov, http:\\maise-guide.org
[11] A. N. Kolmogorov, S. Shah, E. R. Margine, A. K. Kleppe, and A. P. Jephcoat. Pressure-driven evolution of the covalent network in CaB_6. *Physical Review Letters*, 109(7):075501–, 08 2012.
[12] A. N. Kolmogorov, S. Shah, E. R. Margine, A. F. Bialon, T. Hammerschmidt, and R. Drautz. New superconducting and semiconducting fe-b compounds predicted with an \textit{ab initio} evolutionary search. *Physical Review Letters*, 105(21):217003–, 11 2010.
[13] A. F. Bialon, T. Hammerschmidt, R. Drautz, S. Shah, E. R. Margine, and A. N. Kolmogorov. Possible routes for synthesis of new boron-rich fe–b and fe1xcrxb4 compounds. *Applied Physics Letters*, 98(8):081901, 2011.
[14] Dario Alfè. Phon: A program to calculate phonons using the small displacement method. *Computer Physics Communications*, 180(12):2622–2633, 12 2009.
